# Temporal and Spatial Pore Water Pressure Distribution Surrounding a Vertical Landfill Leachate Recirculation Well

**DOI:** 10.3390/ijerph8051692

**Published:** 2011-05-24

**Authors:** Ravi Kadambala, Timothy G. Townsend, Pradeep Jain, Karamjit Singh

**Affiliations:** 1 Department of Environmental Engineering Sciences, University of Florida, P.O. BOX 116450, Gainesville, FL 32611, USA; E-Mails: kadambalar@cdm.com (R.K.); karam@ufl.edu (K.S.); 2 Environmental Engineer III, CDM, 1601 Belvedere Road, Suite 400 East, West Palm Beach, FL 33406, USA; 3 Innovative Waste Consulting Services, LLC, 6628 NW 9th Blvd. Suite 3, Gainesville, FL 32605, USA; E-Mail: pjain@iwcs.biz

**Keywords:** landfill, pore pressure, leachate, piezometers, anisotropy, permeability

## Abstract

Addition of liquids into landfilled waste can result in an increase in pore water pressure, and this in turn may increase concerns with respect to geotechnical stability of the landfilled waste mass. While the impact of vertical well leachate recirculation on landfill pore water pressures has been mathematically modeled, measurements of these systems in operating landfills have not been reported. Pressure readings from vibrating wire piezometers placed in the waste surrounding a liquids addition well at a full-scale operating landfill in Florida were recorded over a 2-year period. Prior to the addition of liquids, measured pore pressures were found to increase with landfill depth, an indication of gas pressure increase and decreasing waste permeability with depth. When liquid addition commenced, piezometers located closer to either the leachate injection well or the landfill surface responded more rapidly to leachate addition relative to those far from the well and those at deeper locations. After liquid addition stopped, measured pore pressures did not immediately drop, but slowly decreased with time. Despite the large pressures present at the bottom of the liquid addition well, much smaller pressures were measured in the surrounding waste. The spatial variation of the pressures recorded in this study suggests that waste permeability is anisotropic and decreases with depth.

## 1. Introduction

Addition of liquids is a common practice at landfills as a method to accelerate waste stabilization, and at times, simply to manage leachate [1]. A variety of methods, including surface application through irrigation or ponds and pressurized injection in buried horizontal trenches or vertical wells, are used to add liquids to the compacted waste [2,3]. A potential concern of the design engineer for such landfills is the impact of an increased hydrostatic pressure in the pore spaces of the waste and cover soil (referred to as *pore pressure*) on the shear strength of the waste mass [4]. A reduction in shear strength by pressurized liquids addition could lead to a slope failure in some cases [5,6].

There is a need for better understanding of how pore pressures build up in landfills as a function of different mechanisms of liquid addition. The impact of liquid addition into vertical wells on pore pressures in the waste has been simulated with mathematical models [7,8]. Simulation results by Jain indicated that even though large pore pressures exist near the well during leachate addition, pressures dissipate within a small radial distance from the well [9]. The objective of the research in this paper was to examine the temporal and spatial impact of leachate recirculation into buried vertical wells on pore water pressure in the surrounding waste in a full-scale municipal solid waste (MSW) landfill. This paper reports experiences of leachate addition in a buried vertical well surrounded by multiple piezometers (pressure transducers) over a 2-year period. Data on spatial distribution of pore water pressure in the surrounding waste in response to cumulative volume of leachate added, and associated leachate injection pressure and operation period, are also presented.

## 2. Experimental Section

### 2.1. Site Description

The study was conducted at the New River Regional Landfill (NRRL) located in Union County, Florida, USA. The NRRL receives approximately 800 metric tons per day of waste consisting of mixed residential and commercial waste. The landfill currently houses several contiguous lined landfill cells totaling approximately 30 hectares. The experiment described here was conducted in Cell 4, a 7.8-hectare cell equipped with a double liner system. The average depth of waste from the surface of the landfill to the leachate collection system at the time when the experiment was conducted was approximately 20 m. The apparent *in situ* bulk density of the landfilled waste was estimated to be approximately 700 kg/m^3^; the apparent *in situ* bulk density was estimated based on the mass of waste deposited in the landfill and the landfill volume estimated by topographic surveys of the site. The density was within the reported range of 300 kg/m^3^ to 2,100 kg/m^3^ for compacted waste [10,11]. A clayey-sandy soil mined on site was used as daily cover. The maximum permitted leachate recirculation rate into Cell 4 was 122 m^3^ per day. The *in situ* saturated hydraulic conductivity for waste deposited in Cell 1 was reported to range from 5.4 × 10^−6^ to 6.1 × 10^−5^ cm/s [12].

### 2.2. Experiment

**Figure 1.**
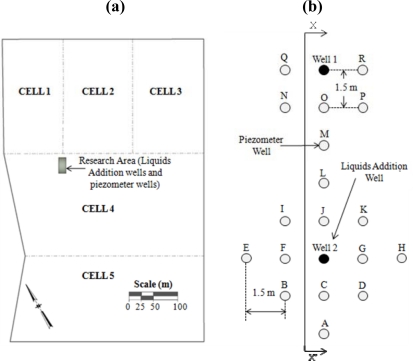
A plan view showing (**a**) the lined cells at New River Regional Landfill (NRRL) and the area of the moisture addition well (Well 2) and the 18 piezometer wells used; and (**b**) the respective locations of the liquids addition well and piezometer wells.

The experiment centered upon a 12-m deep buried vertical well (20-cm diameter, Well 2) augured into the compacted waste at NRRL using a 20-cm hollow stem open flight auger. The screen length of the buried vertical well was 10.5-m from the bottom of the well. Figure 1 shows the location of the experiment with respect to the landfill cells; a second vertical well (Well 1) was installed, but leachate was not added to this well during the experiments reported in this paper.

**Figure 2.**
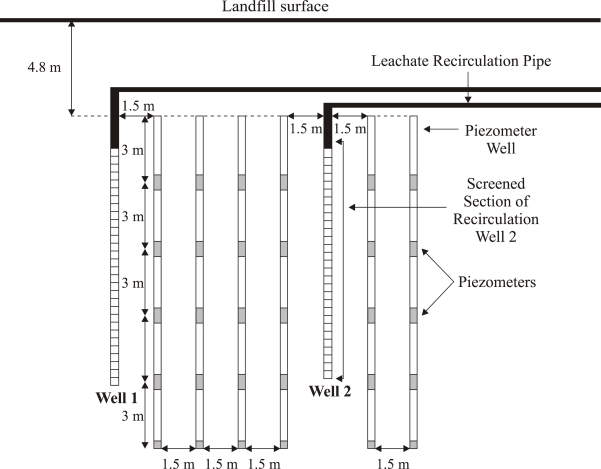
Cross Sectional View of the liquids addition well (Well 2) and the piezometer wells (Cross-section X-X’ in Figure 1).

The well was connected to a lateral polyethylene pipe (7.6 cm in diameter) that extended to the side slope of the landfill, and connected to the main leachate recirculation system. Eighteen multi-level piezometers (Model 52611199, Durham Geo-Slope Enterprises, Mukilteo, WA, USA) were placed 1.5 m away from each other as shown in Figure 1. Each multi-level piezometer consisted of five vibrating wire (VW) piezometers placed three meters apart from each other for a total length of 15 m (Figure 2). The entire multi-level piezometer assembly was placed inside a 20-cm diameter borehole. This bore hole was then grouted with a grout machine (ChemGrout, LaGrange Park, IL, USA) using a mix of cement and bentonite following the piezometer manufacturer instructions. Another 4.8-m lift of waste was placed on top of the experimental area; the vertical wells and the multi-level piezometer wells were buried. All of the piezometers were connected to a datalogger (CR10X, Campbell Scientific, Logan, UT, USA) to record the pore pressure and temperature registered by the buried instruments.

A pressure transducer, pressure gauge, flow meter, and globe valve were installed on each of the two leachate recirculation laterals that individually connected vertical wells to the leachate recirculation header. The leachate injection pressure was measured using a 4–20 mA pressure transducer (GE Druck Inc., Billerica, MA, USA) and read using a loop calibrator (UPS II, GE Druck Inc.). The flow rate and cumulative volume of leachate injected in the buried vertical wells were measured using the SeaMetrics IP80 flow meters (Control Warehouse, Ocala, FL, USA). The globe valves were used to manually control the flow rate of leachate injected in a vertical well.

### 2.3. System Operation and Monitoring

Leachate was recirculated intermittently for a period of approximately four months. The system was operated for the first two months primarily during the operating hours of the facility (8:00 a.m. to 5:00 p.m.) and the experimental parameters (flow rate, injection pressure, cumulative volume through the injection well, and pore pressure and temperature at the piezometer locations) were closely monitored. The system was then continuously (24/7) operated for extended durations (15–20 days) on two occasions during the subsequent two months. The monitoring of the pore pressures in the waste surrounding the wells was continued even after cessation of leachate recirculation. The time of operation, cumulative volume, flow rate, and pressure were recorded every hour manually throughout  the leachate recirculation period (during landfill operating hours). The data logger recorded the pore pressure and temperature from all 90 VW piezometers every hour. Approximately 15–30 m^3^ of leachate was added daily during the operational period and the leachate injection pressure was maintained at a pressure 14–16 m of water column (w.c.) at the bottom of the buried vertical well and the flow rate was maintained at 1.1–1.5 × 10^−3^ m^3^/s.

### 2.4. Data Management

Data from the 90 piezometers were downloaded from the datalogger using LoggerNet 3.X software (Campbell Scientific Software). Pressure data were recorded in frequency (Hz) and the temperature data were recorded in degrees Celsius (°C). The pore pressure data were converted from frequency into units of pressure using the calibration factors and equations provided in the manufacturer’s calibration sheet for each instrument. For easier interpretation of data, the date of calibration of the VW piezometers in the Geokon lab was treated as “day one.” Some of the VW piezometers showed pore pressure as much as 6–8 m of w.c. before leachate injection (apparently from gas buildup). In order to assess the impact of leachate injection into the buried vertical wells on the pore water pressure in the surrounding waste, the measured pore pressures were normalized by subtracting the respective pore pressures from each instrument recorded just prior to injecting leachate for the first time.

## 3. Results and Discussion

### 3.1. Overall Sensor Performance

A total of 58 out of the 90 piezometers worked properly. Thirteen piezometers stopped working immediately after installation and another 19 piezometers either stopped working or started giving highly erratic data over the course of two years. Most of the piezometers that did not work were located in the deeper sections of the multi-level piezometer assemblies. Improper splicing of the wires, clogged piezometer filter caps, and damaged wires due to excessive overburden pressure are potential causes of piezometer malfunction.

The 58 operational piezometers responded to leachate injection by showing an increase in pore water pressure in the surrounding waste. There was an initial time lag of 12–24 hours in reading the correct change in pore pressure as a result of the time required to saturate the grout. However, once the grout became saturated, the piezometers responded within an hour of leachate recirculation. Out of the 58 piezometers that were assessed to be functional, 39 piezometers measured the temperature correctly. The temperature registered by the remaining 19 sensors was outside the calibration range of these transducers (−20 °C to 80 °C), and these were concluded to be malfunctioning.

### 3.2. Pore Pressures in the Waste before Leachate Recirculation

**Figure 3.**
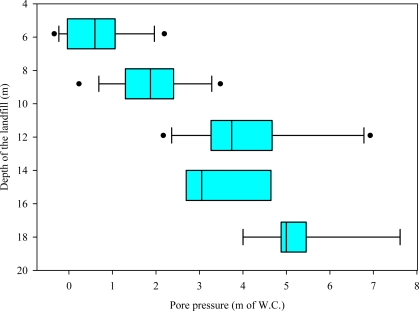
Pore pressures measured by the piezometers at various depths inside the landfill prior to leachate recirculation.

Figure 3 presents the box-and-whisker plot of the pore pressures measured by the piezometers at various depths in the landfill before leachate recirculation. The line inside the box represents the median. The right and left sides of the box represent the 75th percentile and the 25th percentile, respectively. The lines that extend right and left (whiskers) from the box represent the 90th and 10th percentiles, respectively. The outliers are presented individually outside the whiskers as individual data  points. The pore pressures varied from 0.5 m to 5 m of w.c. The initial high pore pressures in the waste are believed to be a result of landfill gas pressure produced from the decomposition of organic waste. The pressure increased substantially with depth.

The grout mix poured in the piezometer wells during installation contained water. The possibility that the high pore pressures were a result of hydrostatic water head in the grout-encased piezometers was examined with a laboratory experiment. The time required for the pore water pressure in the grout to fully dissipate into sand surrounding a grouted piezometer in a laboratory column was approximately 22 days [13]. The high pore pressures were thus concluded not to result from the presence of water in the grout.

To assess whether the landfill gas could be a cause of high pore pressures documented prior to liquids addition, landfill gas pressure at the transducer locations was estimated using the 1-dimensional gas flow model presented by Townsend *et al.* [14]. The results suggested that gas pressures of the magnitude measured in this study were plausible for a landfill with a waste permeability value of k_z_ = 10^−13^ m^2^, which is within the range of permeability values reported for the site [15]. The measured pressures are a combination of leachate and gas pressure; the relative contribution of each of these phases could not be measured in the field and merits further investigation.

### 3.3. Piezometer Responses in the Initial Period of Leachate Recirculation

**Figure 4.**
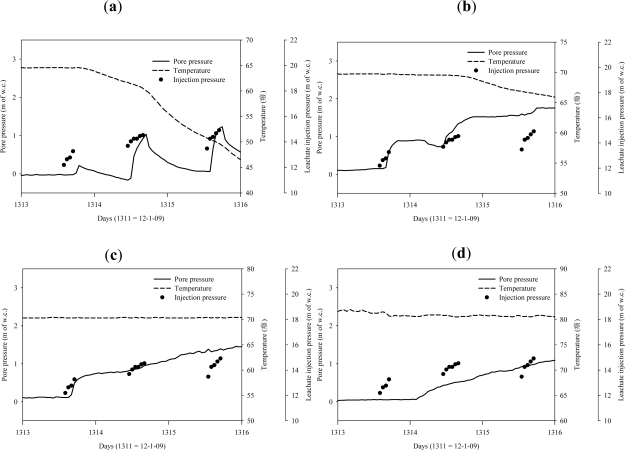
Piezometer responses to leachate recirculation at various depths and at a fixed radial distance of 1.5 m from the well during the first few days of operation at an average flow rate of 1.4 × 10^−3^ m^3^/sec. (**a**) Piezometers at a depth of 5.8 m from the surface of the landfill; (**b**) Piezometers at a depth of 8.8 m from the surface of the landfill; (**c**) Piezometers at a depth of 11.9 m from the surface of the landfill; (**d**) Piezometers at a depth of 18 m from surface of the landfill.

**Figure 5.**
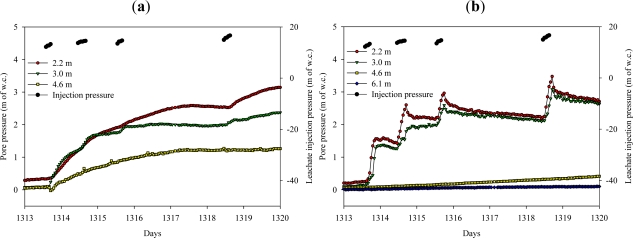
Piezometer responses to leachate recirculation in radial direction from the vertical well during the first few days of operation at an average flow rate of 13.67 × 10^−4^ m^3^/s. (**a**) Piezometers at a depth of 5.8 m from the surface of the landfill; (**b**) Piezometers at a depth of 14.9 m from surface of the landfill.

Several piezometers were selected as examples to illustrate the typical response during the initial days of operation. Leachate recirculation into Well 2 was started on day 1313 and was carried out for 4–6 hours per day as indicated by the leachate injection pressure in Figure 4 and Figure 5. The average flow rate was 1.4 × 10^−3^ m^3^/sec and the applied pressure at the bottom of the well was maintained at 13–17 m of w.c. during the leachate recirculation period.

Figures 4a,b,c,d present the piezometer responses to leachate recirculation at various depths of the landfill and at a fixed radial distance 1.5 m from the vertical well. Figure 4a presents the pore pressure, temperature and leachate injection pressure of a piezometer located at depth of 5.8 m from the surface of the landfill. When leachate recirculation was started on day 1313, the pore pressure increased and the temperature decreased after a lag time of 4 hours when the moisture front reached the piezometers. This lag time is the time taken by the moisture to travel from the well to the piezometer. The temperature decreased for the next several days due to colder temperature of the added moisture, after which it equilibrated with the surrounding waste. The pore pressure increased marginally compared to the leachate injection pressure in the well and quickly decreased within an hour in the first day of operation. In the second day, as soon as the leachate recirculation was started the pore pressure in the waste quickly increased within an hour as the waste was already wetted. When the leachate recirculation was stopped, the pressures in the waste did not drop right away, but slowly decreased with time.

From Figure 4b it is observed that at a depth of 8.8 m, the pore pressure increased gradually within 3 hours of leachate recirculation; however, the temperature did not decrease until the next day. This suggests that the initial increase in pore pressure without an increase in temperature might be due to compression of the landfill gas from the surrounding moisture front movement. In the second day of leachate recirculation, the temperature decreased along with an increase in the pore pressure indicating that the moisture front had reached the piezometer.

From Figure 4c,d it is observed that at deeper locations, the pore pressure increased gradually; however the temperature did not decrease. This indicates that the moisture front might not have reached the piezometers or that the rate of moisture travel was slow enough to allow for temperature equilibration.

The piezometer responses illustrated above were typical of results of the remaining piezometers. The movement of the moisture front was more pronounced in the shallow sections (5.8 and 8.8 m depths) of the landfill compared to the deeper sections (14.9 m and 18 m depths) during the initial days of operation. This is believed to be a result of lower waste permeability in the deeper sections compared to the shallow sections. The trend was not observed in every case, however, as in certain locations the moisture front reached the middle sections of the landfill prior to the shallow sections, possibly a result of preferential pathways for moisture migration due to the heterogeneous nature of the compacted waste.

Figures 5a,b present the piezometer responses to leachate recirculation in the radial direction from the vertical well and at a depth of 5.8 m and 14.9 m from surface of the landfill during the initial days of operation. As expected, the resulting pore pressures decreased with the radial distance from the well due to pressure loss as the moisture traveled through the waste mass.

### 3.4. Temporal Impact of Leachate Recirculation on the Pore Pressure in the Surrounding Waste

A total of 1,422 m^3^ of leachate was recirculated intermittently in the primary buried vertical well (Well 2) for a period of 122 days. The average flow rate of leachate recirculation was 1.4 ± 0.4 × 10^−3^ m^3^/s and the applied pressure at the bottom of the well was 15.38 ± 2.4 m of w.c. Temperature data indicated that the moisture front had wetted 88% of the piezometers; however, most of these wetted piezometers were located up to a radial distance of 2.2 m from the well. One-fourth of the piezometers located at a radial distance of 2.2 m to 7.8 m from the vertical well were not wetted.

**Figure 6.**
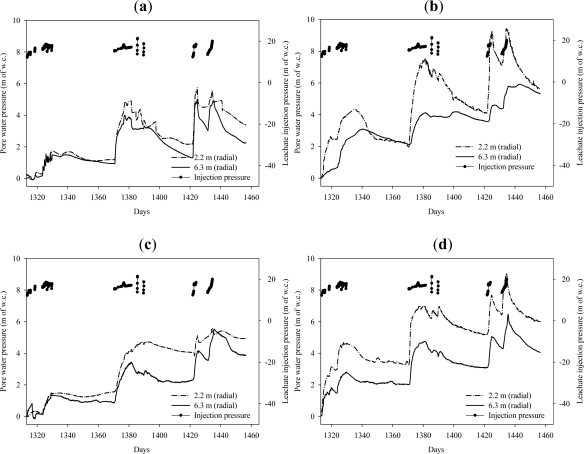

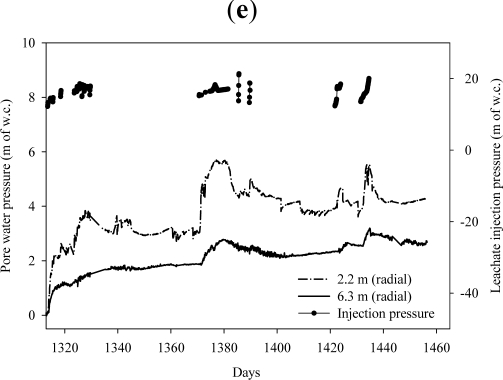
The temporal impact of leachate injection on the pore pressures of surrounding waste in the radial direction and at various depths. (**a**) Piezometers at a depth of 5.8 m from the surface of the landfill; (**b**) Piezometers at a depth of 8.8 m from the surface of the landfill; (**c**) Piezometers at a depth of 11.9 m from the surface of the landfill; (**d**) Piezometers at a depth of 14.9 m from the surface of the landfill; (**e**) Piezometers at a depth of 18 m from the surface of the landfill.

Ten piezometers were chosen to illustrate the temporal impact of liquid recirculation into the buried vertical well on the pore water pressure in the surrounding waste. Five piezometers were located at a radial distance of 2.2 m from the buried vertical well and at a depth of 5.8, 8.8, 11.9, 14.9 and 18 m from the surface of the landfill. The remaining five piezometers were located at a radial distance of 6.3 m from the buried vertical well and at a depth of 5.8, 8.8, 11.9, 14.9 and 18 m from the surface of the landfill. Figure 6 shows the change in pore pressure of the piezometers and the leachate injection pressure over time.

The pore pressures increased steadily to 2–6 m of w.c. over time in all ten piezometers indicating that the degree of saturation in the surrounding waste had increased due to leachate addition. The pore pressure is expected to increase until the steady-state conditions are achieved; based on design charts for a vertical well developed by Jain *et al.* approximately 26,000 m^3^ of leachate need to be added to the well to reach a steady state [16]. This suggests that the pore water pressure will further increase in the surrounding waste with leachate addition as only a small quantity of leachate was added in the vertical well compared to the volume of leachate required to reach the steady state.

The change in pore water pressure with time was higher in all the piezometers located close to the vertical well compared to the piezometers located away from the well at the same depths. This decrease in pore water pressure with the radial distance from the vertical well during leachate recirculation primarily results from a decrease in the hydraulic gradient. The hydraulic gradient reduces due to an increase in the flow path length as the zone of impact increases.

### 3.5. Spatial Impact of Leachate Recirculation on the Pore Pressure in the Surrounding Waste

**Figure 7.**
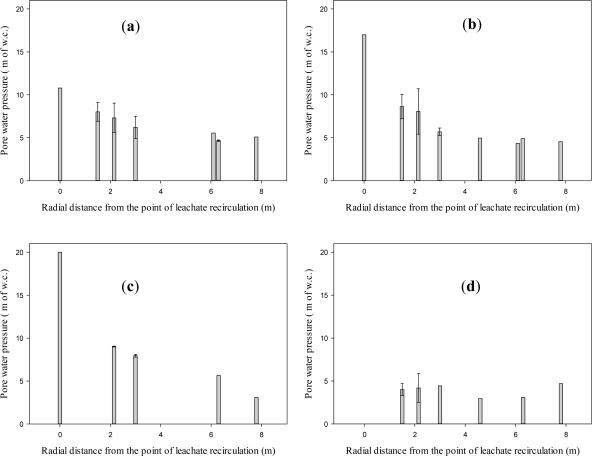
Pore pressure variation in the waste surrounding Well 2 as a function of radial distance on day 93, while the leachate was actively added to the well. Error bars indicate one standard deviation of uncertainty. (**a**) At a depth of 5.8 m from the surface of the landfill; (**b**) At a depth of 11.9 m from the surface of the landfill; (**c**) At a depth of 14.9 m from the surface of the landfill; (**d**) At a depth of 18 m from the surface of the landfill.

**Figure 8.**
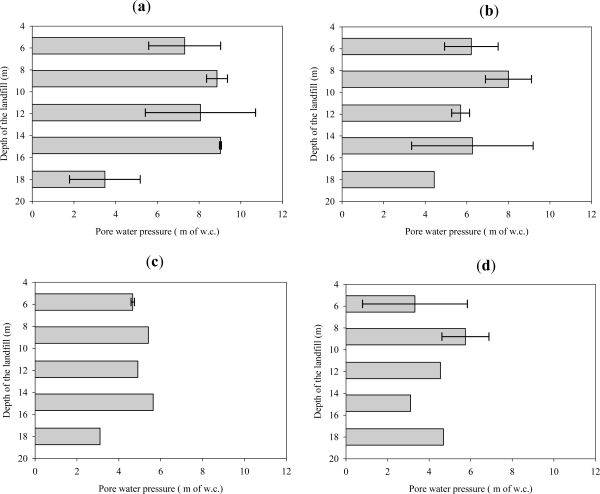
Variation the pore pressures in the waste surrounding Well 2 as a function of depth on day 93 while the leachate was actively added to the well. Error bars indicate one standard deviation of uncertainty. (**a**) A radial distance of 2.15 m from the buried vertical well; (**b**) A radial distance of 3 m from the buried vertical well; (**c**) A radial distance of 6.3 m from the buried vertical well; (**d**) A radial distance of 7.8 m from the buried vertical well.

Figure 7 and Figure 8 present the results of spatial variation of pore pressure in the waste surrounding the leachate recirculation well (Well 2) on day 93, while leachate was actively injected at a pressure of 20 m of w.c. (at the bottom of the well) and at a flow rate of 6 × 10^−4^ m^3^/s. A cumulative volume of 1,400 m^3^ of leachate had been recirculated up to this point of time. As shown in Figure 2, the piezometer wells extended deeper than the leachate recirculation well. Since the pressure at the recirculation well location at a depth corresponding to the bottom of the piezometer well could not be measured, the pressure at the well location for this depth is not presented in Figure 7d.

Figure 7 presents the radial change in pore pressure from the buried vertical well at various depths from the surface of the landfill. In these figures, the pressure at the bottom of the well is shown at a radial distance of zero. Results indicate that the pressures in the waste are much less than the pressures in the well. For example, at a radial distance of 6 m away from the leachate injection well, the pore water pressure reduced by over 50%. The pore water pressure further reduces to 75% with an increase in the depth of the landfill as shown in Figures 7b,c. The results suggest that although large pressure can exist at the well location, the pore pressure dissipates within a short distance from the well. The localized leachate recirculation at high pressure did not result in equally high pressure across the landfill. This observation was in agreement with observation reported by Jain based on numerical modeling [15]. Pore pressure is a key input parameter for evaluating geotechnical stability of landfill slopes. An assumption that the hydrostatic pressure profile everywhere in the landfill will mimic the pressure profile at the well location (a practice of some engineers observed by the authors) as part of the slope stability modeling process may result in an underestimation of factor of safety for slope stability.

At a depth of 18 m, which is 3 m below the bottom of the leachate injection well, the change in pore pressure was significantly lower than the leachate injection pressure at the bottom of the well and was fairly constant in the radial direction away from the buried vertical well as shown in Figure 7d. This supports the generally accepted assessment that compacted MSW is highly anisotropic in nature, with waste permeability in the lateral direction greater than the vertical direction.

Figure 8 presents the change in pore pressure in the vertical direction from the surface of the landfill and at various radial distances from the well. Results indicate that even though the hydrostatic head increased with the depth of the vertical well during leachate injection, the pore water pressure in the waste did not increase proportionally. This trend was similar even at farther locations from the vertical well as shown in Figures 8b,c,d. This is believed to result from several reasons. First, the moisture distribution is not at steady state, but under transient flow conditions; the zone of saturation has not reached the zone of distribution that would be reached under steady state operation [15]. As indicated earlier, the volume of liquids added over the course of this study was less than that needed to achieve the steady-state condition for liquids flow from a vertical well. Second, this observation would follow the previous explanation provided by a decreasing permeability of waste with depth in the landfill. If waste permeability remained constant with depth, increasing pressures deeper in the well would be expected to cause increasing pressures in the surrounding waste as a function of depth.

At a radial distance of 2.15 m from the buried vertical well as shown in Figure 8, there is a significant difference in the pore water pressure in the waste at depths of 14.9 m and 18 m from the surface of the well. Even though the injection pressure was the same and there would be little difference in the flow path length of the leachate between the 14.9 m and 18 m depth, there was a significant difference in the pore water pressures in the waste in these locations. This again can be explained by the combined effect of anisotropy and waste permeability reduction with depth.

## 4. Summary and Conclusions

This paper presents the impact of leachate recirculation into a buried vertical well on the pore pressure in the surrounding waste at a full-scale MSW landfill. After the multi-level piezometers were installed, 58 piezometers worked properly out of the 90 piezometers. This survival rate is typical of harsh conditions such as those encountered in landfills. Before leachate injection, the pore pressures measured by the VW piezometers increased significantly with the depth of the landfill indicating the significant effect of landfill gas generation and a lower permeability of the waste in deeper sections of the landfill.

Fifty-eight piezometers responded to leachate recirculation into the vertical well (Well 2) with an increase in pore water pressure measurements. The shallow piezometers located close to the leachate injection well responded more rapidly to leachate addition compared to piezometers that were deeper and located far from the well. The pressures in the waste did not drop immediately after leachate recirculation was stopped, but rather decreased slowly with time. The piezometers showed a steady increase in pore pressure in the surrounding waste over time and would be expected to continue increasing until leachate addition in the well reached steady state. The thermocouples showed a decrease in temperature when the moisture front reached the piezometers. The temperature decreased for the next several days as a result of the colder temperatures of the added leachate, after which it reached a thermal equilibrium between the moisture and the surrounding waste.

Several observations support that moisture migration and resulting pore pressure distribution were influenced by the anisotropic nature of the waste and the decrease in waste permeability with landfill depth. Even though the hydrostatic head increased with the depth of the vertical well during leachate injection, the pore water pressure in the surrounding waste did not increase proportionally; radial moisture travel was just as great in upper regions of the landfill where hydrostatic pressures were not as great. Pore pressures at depths below the screened portion of the well were often less than pressures at a higher elevation in the landfill, even though the flow paths were relatively equivalent. The observation that large pressures were present in the bottom of the vertical well during leachate addition but much reduced in the surrounding waste supports previous modeling results and should be considered as part of slope stability modeling for landfills with liquids addition. Future efforts will be made to estimate waste hydraulic conductivity and anisotropy by inverse modeling the spatial and temporal pore pressure data reported in this paper.
